# A Predominant Role for Parenchymal c-Jun Amino Terminal Kinase (JNK) in the Regulation of Systemic Insulin Sensitivity

**DOI:** 10.1371/journal.pone.0003151

**Published:** 2008-09-05

**Authors:** Sara N. Vallerie, Masato Furuhashi, Raquel Fucho, Gökhan S. Hotamisligil

**Affiliations:** Department of Genetics and Complex Diseases, Harvard School of Public Health, Boston, Massachusetts, United States of America; University of Bremen, Germany

## Abstract

It has been established that c-Jun N-terminal kinase 1 (JNK1) is essential to the pathogenesis of insulin resistance and type 2 diabetes. Although JNK influences inflammatory signaling pathways, it remains unclear whether its activity in macrophages contributes to adipose tissue inflammation and ultimately to the regulation of systemic metabolism. To address whether the action of this critical inflammatory kinase in bone marrow-derived elements regulates inflammatory responses in obesity and is sufficient and necessary for the deterioration of insulin sensitivity, we performed bone marrow transplantation studies with wild type and JNK1-deficient mice. These studies illustrated that JNK1-deficiency in the bone marrow-derived elements (BMDE) was insufficient to impact macrophage infiltration or insulin sensitivity despite modest changes in the inflammatory profile of adipose tissue. Only when the parenchymal elements lacked JNK1 could we demonstrate a significant increase in systemic insulin sensitivity. These data indicate that while the JNK1 activity in BMDE is involved in metabolic regulation and adipose milieu, it is epistatic to JNK1 activity in the parenchymal tissue for regulation of metabolic homeostasis.

## Introduction

Over the last decade discoveries in the metabolism field, starting with the association of increased tumor necrosis factor alpha (TNF-α) and other inflammatory cytokines in obesity, have demonstrated the strong inflammatory underpinnings of obesity and associated metabolic diseases [1]–[3]. Obesity leads to elevated production of pro-inflammatory molecules such as TNF-α, IL-6, IL-1β, and MCP-1 in experimental murine models and in humans, notably in adipose tissue [4]–[7]. Furthermore, migration of inflammatory cells to obese adipose tissue, particularly at later stages of the disease, may contribute to and possibly propagate inflammatory responses [8], [9]. Alterations in lipids and lipid mediators represent another potential component of both inflammatory responses and insulin resistance in obesity [10]–[14].

Recently, several mechanistic models have been proposed to explain the emergence of inflammatory and stress responses in obesity and type 2 diabetes, including organelle dysfunction influencing mitochondria and endoplasmic reticulum (ER) and associated stress signaling pathways [7], [15], [16]. While it has yet to be determined whether inflammatory and stress signaling pathways are proximal or distal to organelle dysfunction or triggered by peptide or lipid mediators, it is evident that many of these harmful responses have common targets in regulating insulin receptor signaling. One such target is insulin receptor substrate 1 (IRS-1) serine phosphorylation which is mediated by inflammatory kinases such as c-Jun N-terminal kinase (JNK) and IκB kinase beta (IKKβ) and consequently modulates insulin action [17]–[19]. Pharmacological inhibition or genetic ablation of either JNK1 or IKKβ is effective in the treatment of experimental insulin resistance and diabetes [20]–[23].

JNK, a member of the mitogen-activated protein (MAP) kinase family, is activated by a wide variety of stimuli, including cytokines and environmental stress[24]. Previous work by our laboratory and others has shown that JNK1 is necessary for TNF-α induced serine phosphorylation of IRS-1 and insulin resistance in cells and animals [21], [23], [25]. Whole body genetic deficiency of JNK1, but not JNK2, results in marked protection against insulin resistance and hepatosteosis induced by obesity [23]. JNK activity has also been linked to adverse metabolic outcomes in several critical cellular models and tissues. For example, in β-cells of the islet of Langerhans, activation of JNK is involved in the reduction of insulin gene expression and suppression of the JNK pathway protects β-cells against oxidative stress [26]. Additionally, inhibition of JNK activity in liver cells using either dominant negative JNK1 or shRNA against JNK1 lowers circulating glucose and insulin levels and increases insulin sensitivity in obese models [27], [28]. In contrast, JNK1 activity has little effect on muscle glycogen levels or the protein levels of key molecules involved in glucose metabolism, suggesting that enhanced skeletal muscle glucose metabolism may not underlie the direct beneficial effects of JNK1-deficiency in mice [29], [30]. The combined results of these studies reveal that JNK1 activity has differential effects on metabolic disease depending upon tissue and cell type examined.

Adipose tissue inflammation is a critical pathophysiological mechanism underlying obesity-induced metabolic changes [7] and immune cells infiltrate adipose tissue during the late stages of obesity. It remains to be determined, however, whether contributions of bone marrow-derived cells or those of the parenchymal elements are primarily responsible for triggering the inflammatory changes and dictate the detrimental metabolic outcomes of obesity. In the ApoE-deficient model of atherosclerosis, JNK1 activity in the bone marrow-derived cells had no measured effect on metabolic parameters [31]. However, due to the limitations of this model to evaluate insulin action, we examined the role of JNK1 activity in bone marrow-derived elements in development of high fat diet-induced insulin resistance in the C57BL/6 genetic background. Since JNK1 action has been shown to lie at the interface of obesity and inflammation and the results of several studies indicate that macrophage activity may contribute to insulin resistance in diet-induced obesity, we performed bone marrow transplant experiments to test whether myeloid JNK1 regulates the development of insulin resistance. Specifically, we transplanted JNK1-deficient bone marrow into wild type (WT) mice and examined the metabolic impact in the resulting chimeras. We also performed the inverse transplantation experiments to determine the effects of JNK1 activity in the parenchymal tissues on systemic metabolic homeostasis in mice. We conclude that parenchymal JNK1 plays a predominant role in mediating insulin sensitivity in non-immune cells.

## Results

### Regulation of lipid-induced inflammatory responses in macrophages by JNK1 *in vitro*

**Figure 1:**
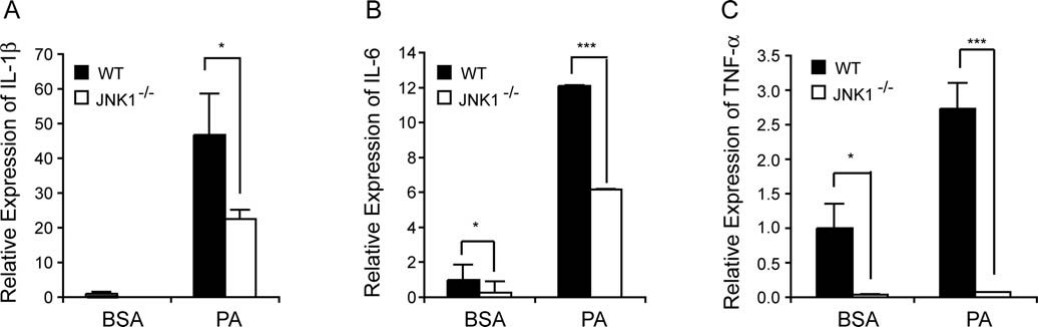
Inflammatory responses in JNK1-deficient macrophages. Primary macrophages isolated from wild type (*WT)* or JNK1-deficient (*Jnk1^−/−^*) mice were stimulated with 250 µM palmitate (PA) for 24 hours. Expression levels of IL-1β, IL-6 and TNF-α mRNAs were determined by real time quantitative RT-PCR and normalized to 18S rRNA. Data are normalized to bovine serum albumin (BSA) treated *WT* macrophages and expressed as mean ± s.e.m. Statistical significance is determined by Student's *t* test, * *p*<0.05 and *** *p*<0.005.

To address the impact of JNK1 activity on the inflammatory capacity of myeloid cells, we examined free fatty acid-induced inflammatory cytokine expression in WT and JNK1-deficient macrophages. For these experiments, we isolated primary peritoneal macrophages from age- and sex-matched *Jnk1^+/+^* and *Jnk1^−/−^* mice and exposed these cells to the saturated fatty acid palmitate. In these cells, we examined the expression levels of IL-6, IL-1β, and TNF-α mRNAs (Fig. 1). Expression levels of these cytokines were significantly decreased in *Jnk1^−/−^* peritoneal macrophages compared to *WT* controls (Fig. 1). These results demonstrate that, under the experimental conditions employed, JNK1-deficiency exhibits a significant impact on the inflammatory output of macrophages upon exposure to palmitate (Fig. 1). These results encouraged us to examine the impact of bone marrow-derived JNK1-deficiency *in vivo*.

### Generation of JNK1-deficient radiation chimeras

**Figure 2:**
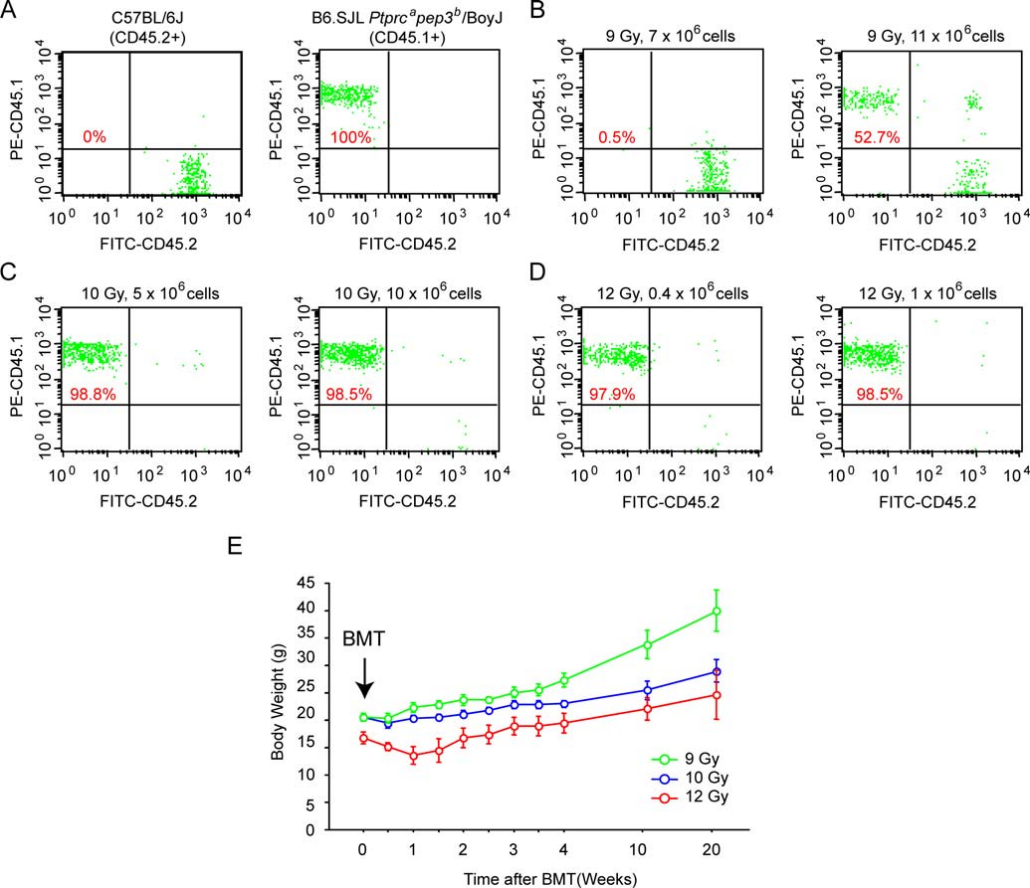
Effects of irradiation dosage on engraftment and body weight. Mice that differ in CD45 cell surface markers were irradiated with 9, 10, and 12 Gy and transplanted with bone marrow (1×10^6^ bone marrow cells) and the rate of chimerism in blood cells were determined by sorting with the specific antibodies for CD45.1 and CD45.2. In an additional control experiment, FACS analysis was performed without BMT using blood cells from C57BL/6J (CD45.2+) and B6.SJL *Ptprc^a^Pep3^b^*/BoyJ (CD45.1+) mice (A). FACS analysis using blood cells from CD45.1→CD45.2 BMT in mice that were irradiated with 9 Gy (B), 10 Gy (C), or 12 Gy (D). Body weight change in the three groups of mice (irradiated with 9, 10, and 12 Gy) following BMT on a regular diet (E). Percentage of reconstitution was calculated and displayed in each graph.

Bone marrow transplantation experiments in genetically modified murine models have been a powerful tool to examine whether immune cells contribute to a given pathology *in vivo.* We initially performed optimization protocols for bone marrow transplantation procedures resulting in efficient reconstitution using B6.SJL *Ptprc^a^Pep3^b^*/BoyJ (CD45.1^+^) and C57BL/6 (CD45.2^+^) mice. We carried out bone marrow transplantation experiments with three different doses of irradiation then determined the ratios of engraftment in blood cells using FACS analysis for CD45.1 and CD45.2 cell surface markers. We also examined body weight changes in the post-transplantation period. These experiments demonstrated that in mice exposed to 10 or 12 Gy of irradiation, the blood cells exhibited more than 98% reconstitution whereas 9 Gy exposure reduced engraftment to 52% (Fig. 2). Mice receiving the lower doses of irradiation gained more weight during the following weeks. Since 10 or 12 Gy irradiation resulted in similar engraftment ratios, we proceeded with the lower dose to reduce the stress and capitalize on the slightly improved recovery and weight gain patterns following transplantation (Fig. 2).

**Figure 3:**
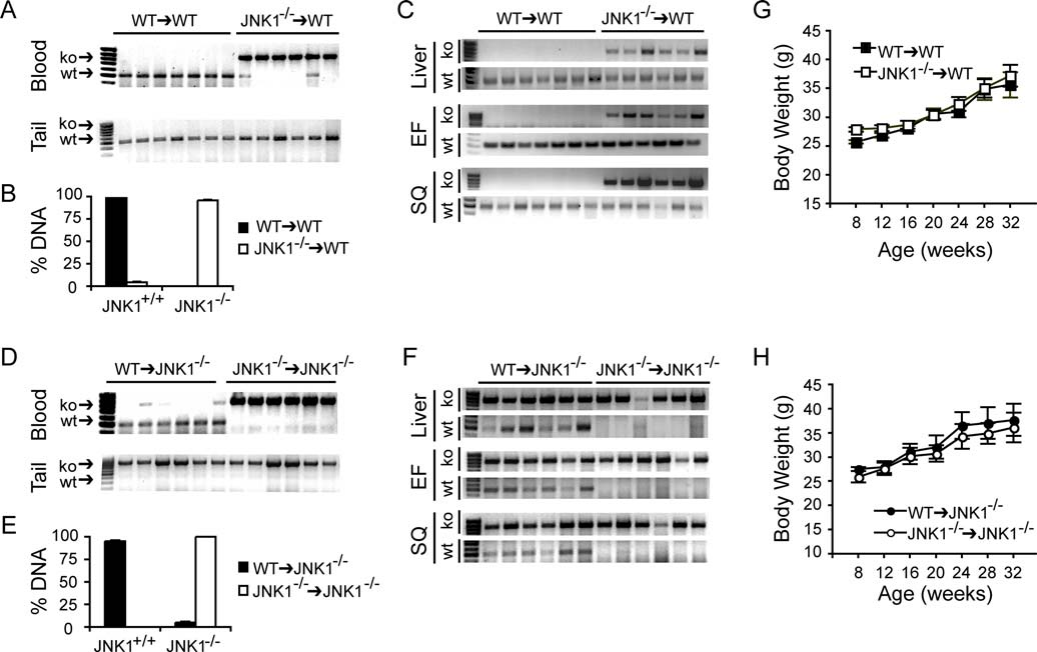
Generation of JNK1-deficient radiation chimeras using bone marrow transplantation. The extent of engraftment of the transplanted cells in *WT* recipients after bone marrow transplantation was determined using genomic DNA isolated from whole blood and quantified with PCR-based allele distribution (A and B). Genomic DNA harvested from tail confirmed the presence of only the recipient genotype (A) while genomic DNA from liver, subcutaneous fat, and epididymal fat tissues (C) confirmed engraftment of donor (*Jnk1*-deficient) cells in *WT* recipients. Genomic DNA isolated from blood was quantified as in panel A except in the *Jnk1^−/−^* recipient groups (D and E). Genomic DNA isolated from the liver, subcutaneous fat, and epididymal fat tissues (F) of *Jnk1^−/−^* recipients confirmed the genotype and engraftment, respectively. At 8 weeks of age, all *WT* (G) and *Jnk1^−/−^* (H) recipient chimeras transplanted with *WT* or *Jnk1^−/−^* bone marrow cells were placed on high fat diet and body weights were monitored for the duration of experiments.

Once an effective bone marrow transplantation procedure was established, this protocol was applied to JNK1-deficient and wild type mice on the C57BL/6 genetic background. To examine the contribution of JNK1 activity in bone marrow-derived cells to insulin sensitivity and metabolic regulation, we created *WT* mice chimeric for myeloid JNK1 expression using bone marrow from *WT* or JNK1-deficient (*Jnk1^−/−^*) donor mice to transplant into *WT* recipients. In this study, 8-week old C57BL/6 male mice were lethally radiated (10 Gy) and transplanted with either *Jnk1^−/−^* (*Jnk1^−/−^*→*WT* experimental group, n = 7) or *WT* (*WT*→*WT* control group, n = 6) bone marrow. As JNK1-deficiency does not yield a significant metabolic phenotype on standard diet, mice were fed a high fat diet starting from 8 weeks up to 35 weeks of age [23]. We determined engraftment by genotyping the blood cells, confirming the successful establishment of the donor genotype and quantified percentage engraftment based on allele distribution (genomic tail DNA served as control, Fig. 3A,B). We also determined the engraftment of the transplanted bone marrow-derived cells in several key recipient sites including liver as well as epididymal and subcutaneous adipose tissues (Fig. 3C). In all tissues examined, the presence of the donor allele in the recipient tissues confirmed the success of transplantation in each experimental animal used in the study. To determine the impact of JNK1 activity in the parenchymal components, we transplanted bone marrow from *WT* or *Jnk1^−/−^* donor mice into JNK1-deficient recipients (*WT*→*Jnk1^−/−^*, n = 6 and *Jnk1^−/−^*→*Jnk1^−/−^*, n = 6) using the same experimental protocol and quantified engraftment in blood cells (genomic tail DNA served as a control, Fig. 3D,E). We also determined the presence of transplanted bone marrow-derived cells in liver as well as epididymal and subcutaneous adipose tissues and confirmed the presence of the donor allele in the recipient tissues and success of transplantation in each experimental animal used in the study (Fig. 3F).

We did not observe any difference in the total body weight of animals in either chimeric recipient groups, *Jnk1^−/−^→WT* and *WT→WT* or *WT→Jnk1^−/−^* and *Jnk1^−/−^→Jnk1^−/−^* groups (Fig. 3G,H). Maximum body weight was approximately 40g, which is lower than what one would achieve in non-transplanted mice that are placed on high fat diet immediately following weaning and without irradiation. These results are consistent with many other reports utilizing transplantation or late placement on diet [32]–[34]. We repeated these experiments in three independent groups and each time the results were essentially identical with no change in body weight between groups (data not shown). In the *WT* recipient experimental group, there was also no statistically significant difference in body weight between the groups and average body weight reached was under 40g. The liver and epididymal fat pad weight was similar between the animals, while percentage of subcutaneous depot was modestly reduced in *Jnk1^−/−^→Jnk1^−/−^* compared to *WT→Jnk1^−/−^* (2.41±0.49 and 4.81±1.14, respectively). These experiments demonstrated that under the experimental protocol employed in our study, none of the chimeric models exhibited a weight regulation phenotype. This lack of difference in body weight allowed a unique advantage to evaluate the impact of bone marrow or parenchymal JNK1-deficiency on systemic insulin sensitivity without the potential confounding effects of weight regulation due to JNK-deficiency.

### Metabolic regulation in JNK1-deficient radiation chimeras

**Figure 4:**
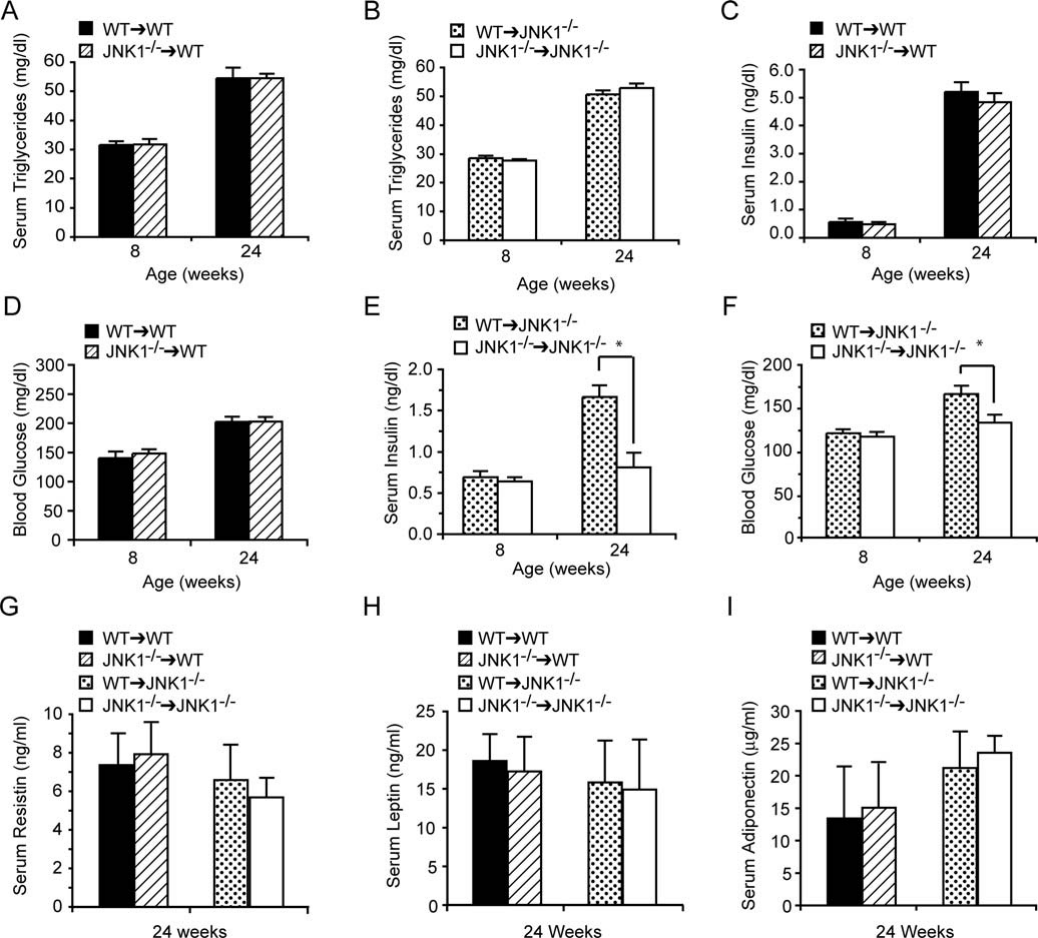
Steady state plasma lipid, glucose, insulin, and serum adipokine concentrations in JNK1-deficient chimeras. Serum samples were collected after a 6 hr food withdrawal from mice from the indicated chimeric groups at 8 and 24 weeks of age. Triglycerides were measured from *WT* (A) and *Jnk1^−/−^* recipients (B) transplanted with either *WT* or *Jnk1^−/−^* bone marrow. Serum insulin (C) and blood glucose (D) levels were measured in the *WT* recipient groups. Serum insulin (E) and blood glucose (F) levels measured in the *Jnk1^−/−^* recipient groups. Serum resistin (G), leptin (H), and adiponectin (I) levels measured in the *WT* and *Jnk1^−/−^* recipient groups at 24 weeks of age. Data are expressed as mean ± s.e.m. Asterisk indicates statistical significance (*p*<0.05) in Student's *t* test.

To evaluate the metabolic status of all chimeras, we determined plasma triglyceride, insulin and glucose levels, and serum adipokines in all animals maintained on high-fat diet. As observed in JNK1-deficient mice, serum lipid levels did not differ in any of the chimeric groups with bone marrow (Fig. 4A) or parenchymal (Fig. 4B) JNK1-deficiency. We also determined plasma insulin and glucose levels in mice that were kept on a high-fat diet for 16 weeks. Among the *WT* recipients, we did not observe any difference in serum insulin (Fig. 4C) or glucose (Fig. 4D) levels between *Jnk1^−/−^*→*WT* and *WT*→*WT* mice. In both groups, serum insulin and glucose levels increased compared to the measurement made at the outset of the study, showing the effect of the dietary intervention in promoting insulin resistance. Interestingly, the serum profiles were significantly different in the *Jnk1^−/−^* recipient chimeras. In this group, *WT*→*Jnk1^−/−^* mice developed relative hyperglycemia and had increased fasting plasma insulin levels compared to *Jnk1^−/−^*→*Jnk1^−/−^* (Fig. 4E,F). Plasma insulin and blood glucose levels in *WT*→*Jnk1^−/−^* chimeras were at intermediate levels between *Jnk1^−/−^*→*Jnk1^−/−^* and *Jnk1^−/−^*→*WT* or *WT*→*WT* groups (Fig. 4C–F). These observations indicated possible protection against insulin resistance when the parenchymal cells lack JNK1 and the potential dominance of this effect on systemic metabolic regulation. Furthermore, we examined serum adipokine levels between the four chimeric groups to determine whether myeloid JNK1 affects adipokines secretion. Analysis of serum resistin, leptin, and adiponectin levels at 8 (data not shown) and 24 weeks of age (Fig. 4G–I) revealed no significant regulation of serum adipokines by either myeloid or parenchymal JNK1-deficiemcy, possibly due to irradiation's effect on weight regulation as previously discussed. These results demonstrate a modest impact of the WT bone marrow-derived cells on the protective phenotype of parenchymal JNK1-deficiency as judged by steady state insulin and glucose measurements indicating the role of bone marrow-derived cells in glucose metabolism.

**Figure 5:**
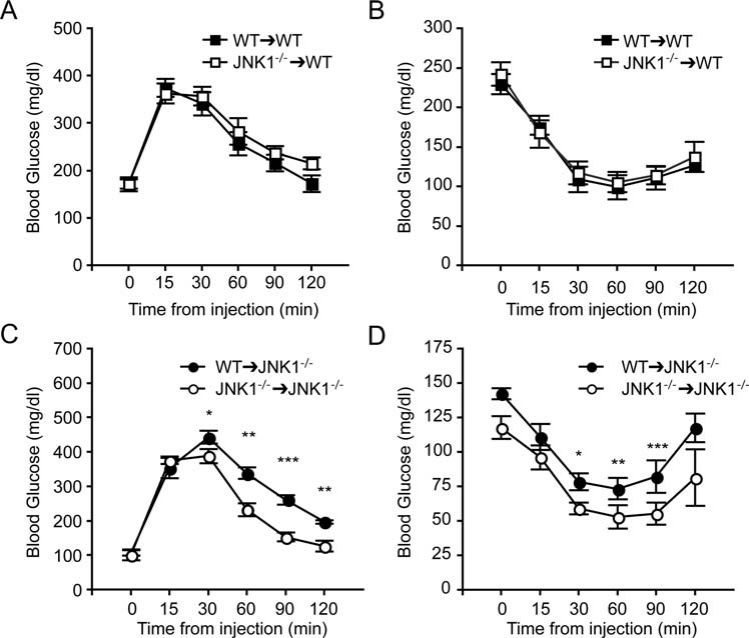
Glucose tolerance and insulin sensitivity in JNK1-deficient chimeras. Glucose (GTT) and insulin (ITT) tolerance tests were performed on mice at age 22 and 26 weeks, respectively. GTT (A) and ITT (B) experiments after intraperitoneal injection of glucose or insulin in *WT* recipient mice transplanted with *WT* or *Jnk1^−/−^* bone marrow. GTT (C) and ITT (D) in *Jnk1^−/−^* recipient mice transplanted with *WT* or *Jnk1^−/−^* bone marrow. All data are presented as mean ± s.e.m. Statistical analysis was performed by Student's *t* test. *p<0.05, **p<0.01, ***p<0.001.

To further investigate systemic glucose metabolism and insulin sensitivity, we performed insulin and glucose tolerance tests in all four groups of animals (Fig. 5). In both tests, results generated from mice with intact JNK1 in the parenchymal cells (*Jnk1^−/−^→WT* and *WT→WT*) were the same regardless of their bone marrow-derived JNK1 activity (Fig. 5A and B), demonstrating that when the JNK1 activity is present in the parenchyma, JNK-deficiency in the bone marrow-derived cells is not sufficient to influence insulin sensitivity. Mice with JNK1-deficient bone marrow activity exhibit enhanced glucose tolerance (Fig. 5C) and insulin sensitivity (Fig. 5D) only when the parenchymal cells are also deficient in JNK1. Taken together, these results indicate that while the principal impact of JNK-deficiency on systemic insulin sensitivity is driven by the parenchymal elements, bone marrow-derived cells also contribute to its total impact, although to a lesser extent.

### Adipose tissue inflammation in JNK1-deficient chimeras

**Figure 6:**
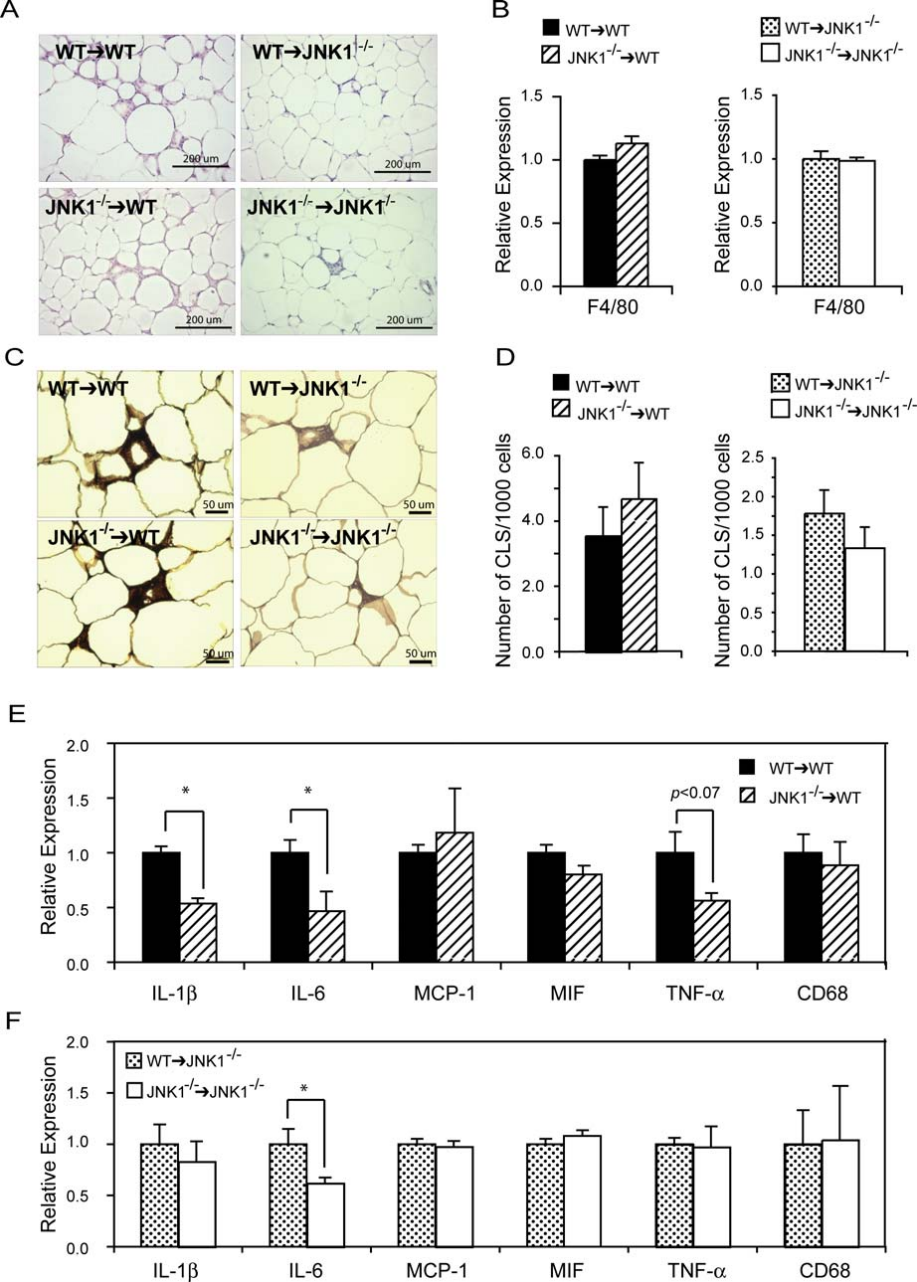
Adipose tissue inflammation in JNK1-deficient chimeras. Photomicrographs of adipose tissue sections were generated after staining with hematoxylin/eosin or anti-F4/80 and hematoxylin. Epididymal adipose tissue sections were prepared from all groups of mice, *Jnk1^−/−^*→*WT, WT→WT, WT*→*Jnk1^−/−^* and *Jnk1^−/−^*→*Jnk1^−/−^*, on high-fat diet for 27 weeks (A). In the same adipose tissue samples, total RNA was extracted and F4/80 mRNA expression level was quantified as an indicator of macrophage infiltration (B). Adipose tissue sections were also stained with anti-F4/80 antibody to detect the protein levels (C) and the number of crown like structures (CLS) were quantified (D). Expression levels of IL-1β, MCP-1, MIF-1, CD68, TNF-α, and IL-6 mRNAs in the epididymal adipose tissue were quantified to determine the cellular milieu in the WT (E) or *Jnk1^−/−^* (F) recipient groups transplanted with *WT* or *Jnk1^−/−^* bone marrow. Asterisk indicates statistical significance (*p*<0.05) in Student's *t* test.

We next examined histological sections from adipose tissues isolated from all groups of animals to determine the effect of myeloid JNK1 on macrophage infiltration and adipocyte morphology. In epididymal adipose depots of *WT* recipient groups, there was a modest increase in mononuclear infiltration compared to *Jnk1^−/−^* recipients regardless of the donor bone marrow type (Fig. 6A). Analysis of F4/80, a macrophage specific marker, showed increased expression in *WT* recipients compared to *Jnk1^−/−^* recipients (0.78 vs. 0.28 AU), but the expression level was not significantly different within the same recipient groups (Fig. 6B). Similar results were also obtained after immunohistochemical analysis of F4/80 protein staining and quantification of crown-like structures in adipose tissue (Fig. 6C,D). These experiments indicated that in either recipient group, the genotype of the donor bone marrow did not influence the degree of mononuclear cell infiltration in chimeric mice but parenchymal *Jnk1^−/−^* mice had reduced signs of inflammation compared to *WT* recipients, regardless of the donor genotype.

Although mononuclear cell infiltration was not affected, we found that JNK1 activity in the bone marrow-derived cells did alter the inflammatory milieu of the adipose tissue. Interestingly, *Jnk1^−/−^→WT* animals had significantly lower IL-6 and IL-1β expression in adipose tissue (Fig. 6E). TNF-α expression trended toward lower, but did not reach statistical significance (*p*>0.07). However, MCP-1, MIF-1, and CD68 expression were not significantly different in the epididymal adipose tissue between *Jnk1^−/−^→WT* and *WT→WT* mice. Similarly, IL-6 levels in *WT→Jnk1^−/−^* chimeric mice were higher than those detected in the *Jnk1^−/−^→Jnk1^−/−^* group (Fig. 6F). In this group the difference in IL-1β levels did not reach statistical significance, and expression of other adipokines, MCP-1, MIF-1, TNF-α and CD68, were not altered between the groups (Fig. 6F). These results demonstrated that while the inflammatory status of the adipose tissue could be moderately altered by bone marrow-derived JNK1 deficiency, this is insufficient to yield a detectable benefit on systemic insulin sensitivity when the parenchymal cells are intact at the JNK1 locus, under the experimental conditions and assays utilized in this study.

**Figure 7:**
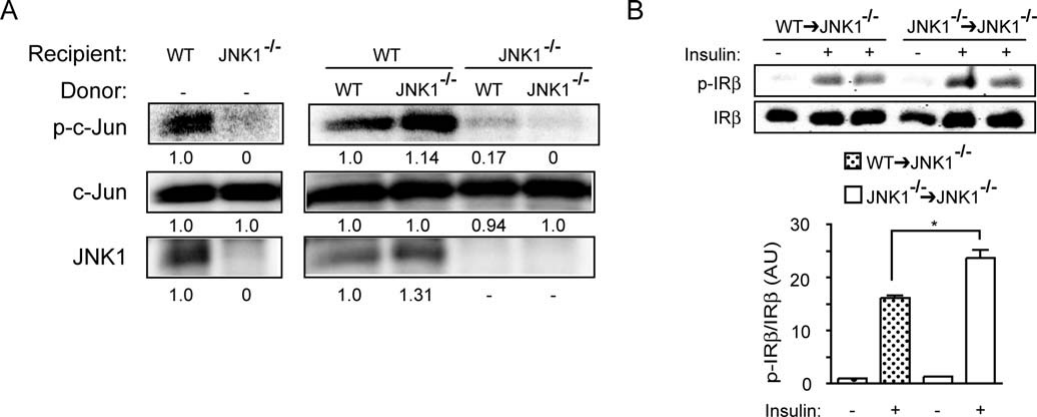
Adipose tissue JNK activity in JNK1-deficient chimeras. Total adipose tissue JNK kinase activity (A) and insulin-stimulated insulin receptor phosphorylation (B) were examined in JNK1-deficient mice transplanted with *WT* or *Jnk1^−/−^* bone marrow. Lower graph in panel B shows the quantification of insulin receptor phosphorylation normalized for insulin receptor protein levels. In the JNK kinase assay, the density of each lane is also shown numerically. Asterisk indicates statistical significance (*p*<0.05) in Student's *t* test.

We also determined JNK1 activity in the adipose tissue of chimeric mice to assess the contribution of parenchyma- and bone marrow-derived elements to obesity related activation of this pathway. In the WT recipient group, there was robust JNK activation in the adipose tissue (Fig. 7A). Since bone marrow-derived cells are a small percentage of total cell population in the adipose tissue, it is not surprising that there was no significant difference in total JNK activity, calculated by the ratio of phosphorylated c-Jun to JNK1 protein, between *Jnk1^−/−^→WT* and *WT→WT* chimeras. The level of JNK activation in the Jnk1*^−^* ^/*−*^ recipient group was significantly lower than the WT recipient group. This result indicates that the main source of obesity-induced JNK activity in adipose tissue is the parenchymal elements such as adipocytes. Nevertheless, in parenchymal JNK1-deficiency, we were able to detect a modest JNK activity in the *WT→Jnk1^−/−^* but not in the *Jnk1^−/−^→Jnk1^−/−^* chimeras, confirming the WT cell deposition in the adipose tissue and the potential impact of bone marrow transplantation. In these mice, we also examined insulin-stimulated tyrosine phosphorylation of the insulin receptor to determine the status of local insulin responsiveness in adipose tissue in intact mice following insulin administration. Insulin-stimulated tyrosine 1162/1163 phosphorylation of insulin receptor beta subunit was modestly decreased in the adipose tissue of *WT→Jnk1^−/−^* mice compared with that of *Jnk1^−/−^→Jnk1^−/−^* controls (Fig. 7B). These results demonstrate that although JNK1 activity in myeloid cells contributes to an altered inflammatory profile and impaired insulin sensitivity in the adipose tissues of Jnk1*^−^* ^/*−*^ mice, this effect is rather modest in magnitude. These results again demonstrated that parenchymal JNK-deficiency is the dominant determinant in the role of obesity-induced metabolic complications.

### Effects of JNK1 activity on liver and subcutaneous adipose tissue

**Figure 8:**
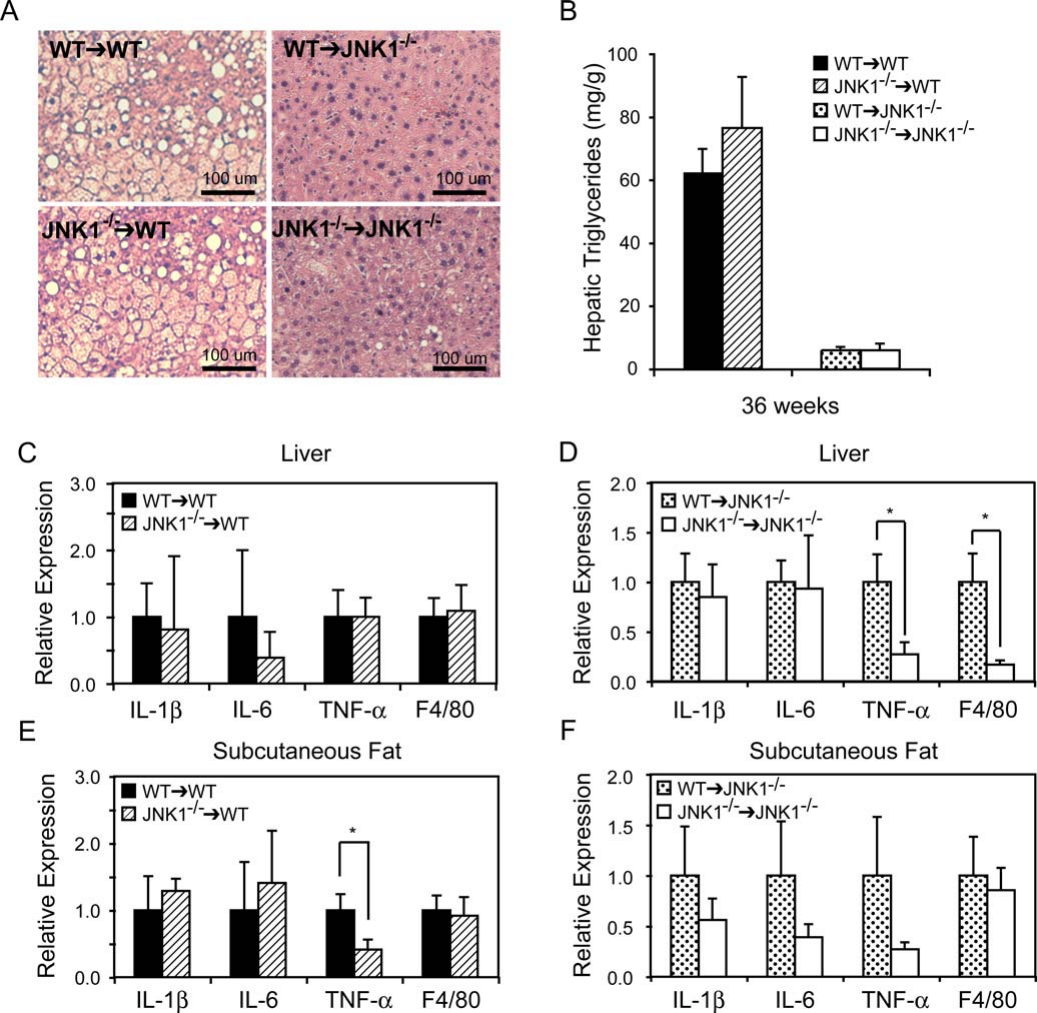
Hepatic triglyceride accumulation and inflammatory cytokines expression in the liver and subcutaneous adipose tissue. Photomicrographs of liver sections were generated after staining with hematoxylin/eosin. Liver sections were prepared from all groups of mice, *Jnk1^−/−^*→*WT, WT→WT, WT*→*Jnk1^−/−^* and *Jnk1^−/−^*→*Jnk1^−/−^*, on high-fat diet for 27 weeks (A). In the same liver samples, triglycerides were extracted and quantified (B). Additionally, total RNA was extracted and expression levels of IL-1β, IL-6, TNF-α, and F4/80 were quantified to determine the cellular milieu in the WT (C) or *Jnk1^−/−^* (D) recipient groups transplanted with *WT* or *Jnk1^−/−^* bone marrow. Similarly, mRNA was extracted from subcutaneous adipose tissue in the WT (E) or *Jnk1^−/−^* (F) recipient groups and subjected to quantitative-PCR analysis of the IL-1β, IL-6, TNF-α, and F4/80 expression. Asterisk indicates statistical significance (*p*<0.05) in Student's *t* test.

Since myeloid JNK1 deficiency did not protect against macrophage recruitment in epididymal adipose tissue, we examined histological sections of liver isolated from all groups to determine the effect of myeloid JNK1 on hepatic triglyceride content. Comparison of liver sections revealed that there was a strong association of lipid accumulation with recipient group (Fig. 8A), similar to the observation of macrophage recruitment. Indeed, in the *WT* recipient groups there was an increase in hepatic triglyceride content compared to *Jnk1^−/−^* recipients groups (68.6±7.7 mg/g vs. 5.98±1.06 mg/g, respectively, *p*<0.0001), although there was no significant difference within the chimeric groups (Fig. 8B).

We further investigated the liver phenotype to determine whether bone marrow derived elements affected the inflammatory state of the liver. In the *WT* recipient group, we were unable to detect any alterations in IL-1β, IL-6, TNF-α, and F4/80 expression (Fig. 8C) between the donor types. Interestingly, *WT→Jnk1^−/−^* animals had statistically significant increase in hepatic TNF-α and F4/80 expression (Fig. 8D). However, analysis of IRS-1, IR, and Akt phosphorylation did not show alterations in the insulin signaling pathway between the two groups (data not shown). We also examined the inflammatory milieu of the subcutaneous adipose depot, which is often different from that of the epididymal adipose tissue when animals are placed on high fat diet. In the *WT* recipients, JNK1-deficiency in the BMDE resulted in lower TNF-α expression (Fig. 8E). Other cytokines examined were unchanged. In contrast, the *Jnk1^−/−^* recipients had no statistically significant alterations in cytokine expression in the subcutaneous tissue (Fig. 8F). In all groups and tissues tested, MCP-1 and CD68 expression levels were unchanged within the groups (data not shown). These results suggest that myeloid JNK1's regulation of cytokine expression is tissue specific and that parenchymal JNK1 activity may influence myeloid JNK1's effects.

## Discussion

Previous studies have demonstrated that whole body JNK1-deficiency results in protection from genetic and diet-induced obesity and insulin resistance [23]. Recently, the critical role of liver JNK activity was also demonstrated by linking liver-restricted activation or suppression of JNK1 to strong systemic metabolic regulation, indicating that this site is critical in the role of JNK in insulin resistance [27]. A similar observation was made for another inflammatory kinase, IKKβ, where its liver-restricted activation or suppression was sufficient to alter systemic insulin action [35]. Genetic interference with regulators of inflammatory pathways, such as IKKβ and PPARγ, in myeloid derived cells has also been demonstrated to confer partial protection from insulin-resistance [32], [35], [36]. Our studies show that JNK1 activity in myeloid cells influences the cellular milieu of the adipose tissue and liver as well. Interestingly, however, the effect of myeloid JNK1 on the inflammatory cytokine expression is dependent on the tissue type examined. When JNK1 is present in the parenchyma, JNK1-deficieny in the bone marrow-derived cells does not confer protection against diet-induced insulin resistance, despite detectable but modest effects on adipose tissue inflammation. We were able to detect a small, but significant, negative impact of *Jnk1^+/+^* macrophages on *Jnk1^−/−^* recipients. These results indicate that JNK1 activity in myeloid cells is not sufficient to alter systemic glucose metabolism and that parenchymal JNK activity is the dominant determinant for metabolic homeostasis.

Furthermore, our results also suggest that conditions that alter the inflammatory gene expression in bone marrow-derived cells would not always yield corresponding changes in systemic insulin sensitivity. In this sense, the metabolic status of the parenchymal targets such as adipocytes or hepatocytes appears to be the driver of both inflammatory alterations and their systemic metabolic consequences. Although similarly identifying a role for JNK1 outside the parenchyma, a recent work by another group came to somewhat different conclusions regarding the magnitude of the contributions of JNK activity in the bone marrow-derived elements on systemic insulin action, where JNK1-deficiency in the bone marrow was sufficient to improve insulin sensitivity even in the WT recipients [37]. In this study, the disease in general appeared to be more advanced despite a later start of dietary interventions, manifesting in higher average body weights and more severe disease. Hence, the differences in our results can be explained by the differences in bone marrow transplantation protocols (early vs. late dietary invention and the timing of the transplantation) and the magnitude of the disease achieved through high fat diet exposure. It is possible that at later stages of the disease, massive degeneration of tissue and recruitment of higher numbers of macrophages could exhibit a larger impact on general metabolism through the action of these bone marrow-derived cells, as reported [37].

In this study, we did not observe any changes in total body weight between the chimeric groups, whereas without transplantation, JNK1-deficiency results in a modest reduction in adiposity on a high fat diet [23]. This difference in body weight patterns is most likely due to the irradiation and delayed exposure time to diet which results in milder weight gain as reported in many previous studies [34]. In fact, irradiation prevented increased adiposity even in the ob/ob model of severe genetic obesity, as recently reported by Ablamunits, *et al.,* but did not interfere with the development of insulin resistance [33]. This is consistent with our observations where we did observe the development of insulin resistance with differences in body weight between groups which allowed examination of the impact of JNK activity independent of secondary effects of body weight and adiposity. At this early stage disease model, the parenchymal cells such as adipocytes and hepatocytes appear to drive the initial events impacting insulin sensitivity and glucose metabolism. The ability to modulate systemic insulin sensitivity through manipulation of JNK1 action in the liver also supports this inference [27], [28]. It would be interesting to determine whether a similar impact could be generated by adipocyte-specific alterations in JNK activity in mice.

Nonetheless, our results demonstrate that myeloid JNK1 plays a role in the regulation of cytokine expression in adipose tissue and the development of insulin resistance in type 2 diabetes, however, it does not appear to be the driver of this phenotype at earlier stages of obesity and insulin resistance, supporting the concept that metabolic target cells are likely to provide the initial inflammatory and/or stress signals that result in metabolic deterioration and their involvement is paramount to metabolic disease.

## Materials and Methods

### Macrophage studies

Primary macrophages were harvested from *Jnk1^+/+^* or *Jnk1^−/−^* mice for analysis of alterations in gene expression as previously described [13]. Isolated primary monocytes were plated in RPMI 1640 (Invitrogen, Carlsbad, CA) with 10% fetal bovine serum (Hyclone, Logan, UT), 1% penicillin-streptomycin (Invitrogen) for 24 hr at 10^4^ cells/well in 6-well plates. Next day, macrophages were treated with 0.5% bovine serum albumin (BSA) or 0.5 mM palmitate in 0.5% BSA in RPMI for 24 hr. All of the procedures in this study were in accordance with Institutional Animal Care and Use Committee of Harvard University, Cambridge, MA.

### Generation of radiation chimeras, transplantation protocols, and fluorescence-activated cell sorter (FACS) analysis

Development and characterization of *Jnk1^−/−^* mice were described elsewhere [38]. B6.SJL *Ptprc^a^Pep3^b^*/BoyJ (CD45.1^+^) and C57BL/6 (CD45.2^+^) mice were obtained from Jackson Laboratory. Bone marrow was collected from donor mice (10–12 weeks of age) by flushing femurs with phosphate buffered saline (PBS). Recipient mice (8 weeks old) were lethally irradiated (9, 10, or 12 Gy) by a cesium gamma source. Four hours later, 5–10×10^6^ bone-marrow cells in 0.3 ml volume were transplanted by intravenous injection, as described[39]. One-week prior and 2-weeks post-bone marrow transplantation, 100 mg/L neomycin and 10 mg/L polymyxin B sulfate (Sigma-Aldrich, St. Louis, MO) were added to the acidified water. Fluorescence-activated cell sorter (FACS) analysis was performed using peripheral blood cells collected from recipient mice 4 weeks after the bone marrow transplantation and staining with antibodies against the cell-surface markers, PE-CD45.1 and FITC-CD45.2 (eBioscience, San Diego, CA). Following incubation with the primary antibodies, FACS lysing solution was added, cells were centrifuged at 1000 g for 2 min, resuspended in 1 ml FACSflow fluid and analyzed on a FACSCalibur apparatus using CellQuest software (all BD Bioscience, San Jose, CA).

### Genotyping

Genomic DNA was harvested from whole blood as described [40] and resuspended in dH_2_0. Percentage of engraftment was evaluated by the allelic ratios. Genomic DNA from liver, subcutaneous fat, and epididymal fat was isolated with DNeasy tissue isolation system (Invitrogen) for use in PCR-based genotyping of wild type and JNK1-deficient alleles as previously described [38].

### Metabolic studies

Male mice were housed in a pathogen-free facility and placed on a high-fat/high-caloric diet (D12492: 60% kcal% fat; Research Diets, Brunswick, NJ) ad libitum starting at 8 weeks of age and were followed for a period of up to 36 weeks. Body-weight measurements were taken starting at 8 weeks of age. Blood samples were collected after a 6-h daytime fast at indicated ages. Serum glucose, insulin, and triglyceride levels were measured as described [2], [23]. Serum levels of resistin (Millipore, Billerica, MA), leptin (Alpco, Salem, NH), and adiponectin (Alpco, Salem, NH) were measured following the provided protocol. Glucose and insulin tolerance tests were performed on conscious mice after a 14-h or 6-h fast, respectively, as described [2], [12], [23], with intraperitoneal administration of glucose (1.5 g/kg, 22 weeks) or human insulin (1 U/kg, 26 weeks; Lilly Research Laboratories, Indianapolis). Triglycerides were extracted from the liver using Bligh and Dryer method [41] and quantified using Serum Triglyceride Determination Kit (Sigma-Aldrich).

### Tissues and gene expression

Total RNA was isolated from epididymal adipose tissue, subcutaneous adipose tissue, liver, or primary macrophages using TRIzol reagent and protocol (Invitrogen). The reverse-transcription reaction was carried out with the high capacity cDNA reverse transcription system (Applied Biosystems, Foster City, CA). Real-time quantitative PCR analysis was performed in a 25-µl final volume using SYBR green PCR master mix (Applied Biosystems). The thermal cycling program was: 10 min at 95°C, 40 cycles, 15 s at 95°C, 30 s at 58°C, and 30 s extension at 72°C. PCR products were confirmed by melting curve analysis. Quantifications were normalized to the 18S rRNA level in each reaction. Primer sequences were as reported previously [12] except MIF with forward GCCAGAGGGGTTTCTGTCG, and reverse GTTCGTGCCGCTAAAAGTCA sequences. JNK kinase assays and insulin receptor phosphorylation experiments were performed as previously described [12].

### Histological analysis

Following sacrifice, a portion of the epididymal adipose tissue and liver were placed in 10% formalin for 24 hours. Tissue samples were paraffin embedded and 5 µm serial sections were prepared from each sample. Samples were deparaffinized, washed three times and then stained with either H&E and mounted or incubated with anti-F4/80 and washed. After PBS washes, sections were incubated with Vectastain ABC-AP Rat IgG Kitand visualized with ImmPACT DAB Substrate Kit (all Vector Laboratories, Southfield, MI). Sections were viewed under a microscope at ×10 and ×40 magnification and photomicrographs were generated with an Olympus CKX41 camera to capture the presence of macrophages in the epididymal adipose tissue. Data are reported as number of total adipocytes surrounded by F4/80+ cells per 1000 adipocytes. Samples from at least 5 mice per genotype were incorporated into the analysis.

### Statistical Analyses

Experimental results were shown as the mean ± s.e.m. Differences between two sets of data were compared by Student's *t* test. All statistical tests with *p*<0.05 were considered significant.
