# Correction: A Predominant Role for Parenchymal c-Jun Amino Terminal Kinase (JNK) in the Regulation of Systemic Insulin Sensitivity

**DOI:** 10.1371/annotation/1074cd39-708b-45ef-943a-e9d1c5bbdedd

**Published:** 2009-01-20

**Authors:** Sara N. Vallerie, Masato Furuhashi, Raquel Fucho, Gökhan S. Hotamisligil

In Figure 8A, the top and the bottom left-hand panels depict an erroneous duplication of the same photomicrograph. Please view the corrected version of Figure 8 here: 

**Figure 8:**
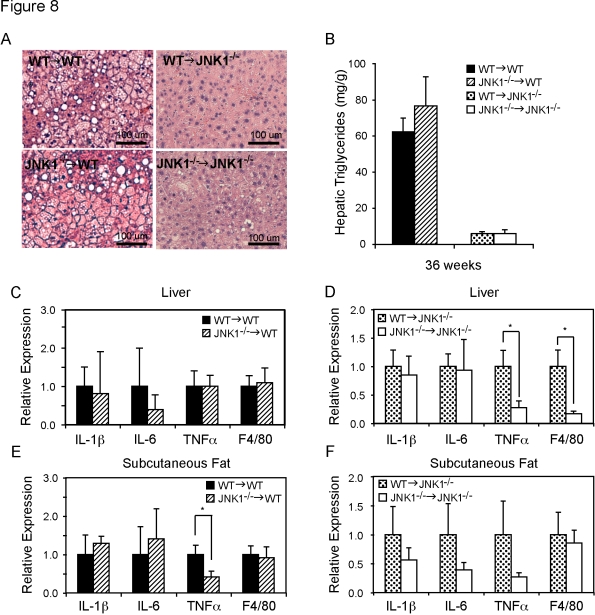
Hepatic triglyceride accumulation and inflammatory cytokines expression in the liver and subcutaneous adipose tissue. Photomicrographs of liver sections were generated after staining with hematoxylin/eosin. Liver sections were prepared from all groups of mice, *Jnk1^−/−^*→*WT, WT→WT, WT*→*Jnk1^−/−^* and *Jnk1^−/−^*→*Jnk1^−/−^*, on high-fat diet for 27 weeks (A). In the same liver samples, triglycerides were extracted and quantified (B). Additionally, total RNA was extracted and expression levels of IL-1β, IL-6, TNF-α, and F4/80 were quantified to determine the cellular milieu in the WT (C) or *Jnk1^−/−^* (D) recipient groups transplanted with *WT* or *Jnk1^−/−^* bone marrow. Similarly, mRNA was extracted from subcutaneous adipose tissue in the WT (E) or *Jnk1^−/−^* (F) recipient groups and subjected to quantitative-PCR analysis of the IL-1β, IL-6, TNF-α, and F4/80 expression. Asterisk indicates statistical significance (*p*<0.05) in Student's *t* test.

